# 2,9,16,19,22,25-Hexaoxatetra­cyclo­[24.4.0.2^4,7^.0^10,15^]dotriaconta-1(26),4,6,10(15),11,13,27,29,31-nona­ene

**DOI:** 10.1107/S1600536809035399

**Published:** 2009-09-09

**Authors:** Jai Young Lee, Ji-Eun Lee, Wonbo Sim, Ki-Min Park

**Affiliations:** a Department of Chemistry, Konyang University, Nonsan 320-711, Republic of Korea; b Central Instrument Facility, Gyeongsang National University, Jinju 660-701, Republic of Korea; c Research Institute of Natural Science, Gyeongsang National University, Jinju 660-701, Republic of Korea

## Abstract

The title 22-crown-6 unit, C_26_H_28_O_6_, comprising of three benzo groups and triethyl­ene glycol, was prepared by the reaction of α,α′-dibromo-*p*-xylene with 1,8-bis­(2-hydroxy­phen­oxy)-3,6-dioxaoctane in the presence of Cs_2_CO_3_ with tetra­hydro­furan (THF) and recrystallized from dichloro­methane–hexane (1:20 *v*/*v*) at room temperature. In the mol­ecular structure, two O atoms of the central ethyl­ene glycol in the triethyl­ene glycol unit exhibit *exo* conformations due to intra­molecular C—H⋯O inter­actions. A number of C—H⋯O and C—H⋯π inter­molecular inter­actions contribute to the stabilization of the crystal packing.

## Related literature

For the preparation of related compounds, see: Sim *et al.* (2001 ▶); Weber & Vögtle (1976 ▶). For a related structure, see: Sim *et al.* (2001 ▶). For background to crown ether-based macrocyclic compounds and their inclusion behaviour, see: Gokel & Korzeniowski (1982 ▶); Izatt & Christensen (1981 ▶); Lindoy (1989 ▶); Pedersen (1967 ▶); Vögtle & Weber (1985 ▶); Weber *et al.* (1989 ▶); Wolf *et al.* (1987 ▶).
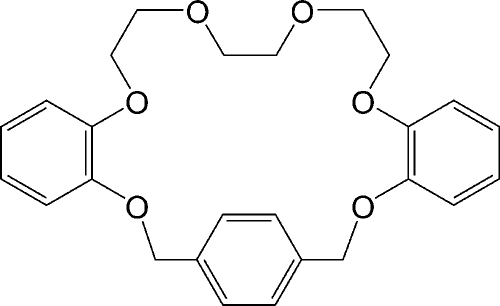


## Experimental

### 

#### Crystal data

C_26_H_28_O_6_
*M* *_r_* = 436.48
Monoclinic, 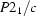

*a* = 12.348 (3) Å
*b* = 18.908 (4) Å
*c* = 9.824 (2) Å
β = 105.70 (3)°
*V* = 2208.0 (8) Å^3^
*Z* = 4
Mo *K*α radiation
μ = 0.09 mm^−1^
*T* = 293 K
0.25 × 0.20 × 0.10 mm

#### Data collection

Bruker SMART CCD area-detector diffractometer
Absorption correction: none
4142 measured reflections
3896 independent reflections
2654 reflections with *I* > 2σ(*I*)
*R*_int_ = 0.057

#### Refinement

*R*[*F* ^2^ > 2σ(*F* ^2^)] = 0.041
*wR*(*F* ^2^) = 0.116
*S* = 1.02
3896 reflections
289 parameters
H-atom parameters constrained
Δρ_max_ = 0.14 e Å^−3^
Δρ_min_ = −0.19 e Å^−3^

### 

Data collection: *SMART* (Bruker, 2000 ▶); cell refinement: *SAINT-Plus* (Bruker, 2000 ▶); data reduction: *SAINT-Plus*; program(s) used to solve structure: *SHELXTL* (Sheldrick, 2008 ▶); program(s) used to refine structure: *SHELXTL*; molecular graphics: *SHELXTL* and *DIAMOND* (Brandenburg, 1998 ▶); software used to prepare material for publication: *SHELXTL*.

## Supplementary Material

Crystal structure: contains datablocks I, global. DOI: 10.1107/S1600536809035399/jh2101sup1.cif

Structure factors: contains datablocks I. DOI: 10.1107/S1600536809035399/jh2101Isup2.hkl

Additional supplementary materials:  crystallographic information; 3D view; checkCIF report

## Figures and Tables

**Table 1:** Hydrogen-bond geometry (Å, °)

*D*—H⋯*A*	*D*—H	H⋯*A*	*D*⋯*A*	*D*—H⋯*A*
C9—H9*A*⋯O2	0.97	2.62	3.114 (2)	112
C10—H10*A*⋯O5	0.97	2.70	3.117 (2)	106
C2—H2⋯O1^i^	0.93	2.76	3.471 (2)	134
C7—H7*B*⋯O5^ii^	0.97	2.72	3.607 (2)	153
C11—H11*B*⋯O1^ii^	0.97	2.82	3.501 (3)	128
C11—H11*B*⋯O2^ii^	0.97	2.90	3.798 (3)	154
C12—H12*B*⋯O4^iii^	0.97	2.42	3.294 (3)	149
C25—H25⋯O3^ii^	0.93	2.71	3.433 (2)	135
C12—H12*A*⋯*Cg*1^ii^	0.97	2.76	3.47	138
C21—H21⋯*Cg*2^i^	0.93	2.97	3.47	115
C26—H26*A*⋯*Cg*3^i^	0.97	3.06	3.83	138

Symmetry codes: (i) 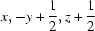; (ii) 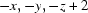; (iii) 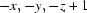. *Cg*1, *Cg*2 and *Cg*3 are the centroids of the C1–C6, C13–C18 and C20–C25 rings, respectively.

**Fig. 1.**
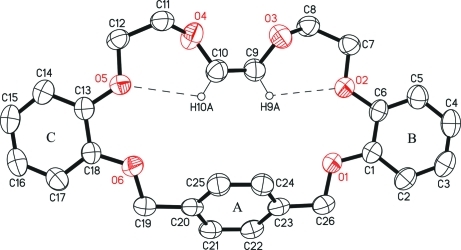
ORTEP drawing of (II) with the atom-numbering scheme and intramolecular C—H···O interactions (dotted lines). Displacement ellipsoids are drawn at the 50% probability level. All H atoms except two H atoms related to intramolecular C—H···O hydrogen bonds have been omitted for clarity.

**Fig. 2.**
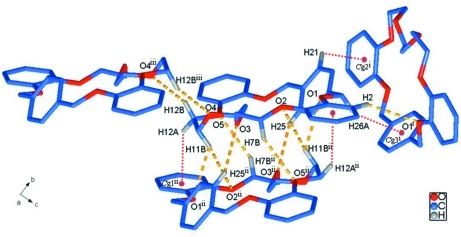
Intermolecular C—H···π (red dotted lines) and C—H···O (yellow dotted lines) interactions in the title compound. All H atoms except those related to intermolecular interactions have been omitted for clarity. Cg1, Cg2 and Cg3 denote the centroids of rings consisting of C1/C2/C3/C4/C5/C6, C13/C14/C15/C16/C17/C18 and C20/C21/C22/C23/C24/C25, respectively. [Symmetry codes: (i) x, -y + 1/2, z + 1/2; (ii) -x, -y, -z + 2; (iii) -x, -y, -z + 1]

**Fig. 3.**
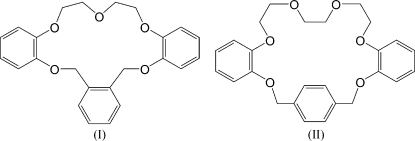
The structures of (I) and (II).

## supplementary crystallographic information

### Comment

An extraordinary variety of crown ether-based macrocyclic compounds have been synthesized and reported since crown ether was first discovered (Pedersen, 1967) because their topological interesting as well as their inclusion behaviour (Gokel & Korzeniowski, 1982; Izatt & Christensen, 1981; Lindoy 1989; Vögtle & Weber, 1985; Weber *et al.* 1989). We have also synthesized and reported the preparation and the solid-state structure of new crown ether (I) bearing three aromatic subunits (Sim *et al.*, 2001). As a part of our continuing interest in the development of new crown compounds, the preparation and crystal structure of new crown ether-based macrocyclic compound (II) containing three benzo units of which ring size is larger than that of the previous reported compound (I) are presented here.

The title compound (II), 2,9,16,19,22,25-hexaoxatetracyclo[24.4.2^4,7^.0.0^10,15^]-dotriaconta-1(26),4,6, 10 (15),11,13,27,29,31-nonaene was prepared by the reaction of α,α'-dibromo-*p*-xylene with 1,8-bis(2-hydroxyphenoxy)-3,6-dioxaoctane in the presence of Cs_2_CO_3_ with tetrahydrofuran (THF) and recrystallized from dichloromethane/hexane (1:20) at room temperature to give colorless single crystals suitable for X-ray analysis.

In the molecular structure of (II), the torsion angels of C—C—O—C connecting A-to-B rings and A-to-C rings aromatic are 167.0 (2)° and 163.4 (2)°, respectively, which indicate that the A ring is situated *trans* to both the B and C rings, with dihedral angles of 57.04 (8)° between A and B and 44.41 (8)° between A and C. The dihedral angle between B and C rings is 14.2 (1)°. The all O—C—C—O and C—C—O—C torsion angles except two C—C—O—C in the triethylene glycol group exhibit *gauche* conformation. Two exceptional C—C—O—C (C10—C9—O3—C8 and C9—C10—O4—C11) torsion angles are *trans* conformation with the values of -162.5 (2)° and -156.1 (2)°, respectively.

Interestingly, two oxygen atoms of the central ethylene glycol in the triethylene glycol unit exhibit the *exo*-orientations which are very different from those found in the ethylene glycol backbone of (I) (Sim *et al.*, 2001) or common crown ether-based compounds. In general, oxygen atoms of ethylene glycol group in crown ether-based compounds favor *endo*-orientation (Wolf *et al.*, 1987). The *exo*-orientations of two oxygen atoms (O3 and O4) in (II) may be due to the intramolecular C—H···O interactions; 2.62 Å for C9—H9A···O2 and 2.70 Å for C10—H10A···O6 (Fig. 1 & Table 1).

The crystal packing is stabilized by not only intermolecular C—H···O hydrogen bonds with C—H···O separation in the range of 2.71–2.90 Å but intermolecular C—H···π interactions with C—H···*Cg* separations in the range of 2.76–3.06 Å (Fig. 2 & Table 1; *Cg* is the centroid of aromatic ring).

### Experimental

To a refluxing suspension of caesium carbonate (15.2 mmol) in THF under nitrogen was added dropwise a solution of α,α'-dibromo-*p*-xylene (3.75 mmol) and 1,8-bis(2-hydroxyphenoxy)-3,6-dioxaoctane (3.79 mmol) in THF over a period of 3 h. The mixture was then refluxed for an additional 24 h. After cooling to room temperature, 10% aqueous hydrochloric acid was added. The solvent was removed under reduced pressure and the residual mixture was extracted with dichloromethane. The organic layer was washed with water, dried over anhydrous magnesium sulfate, and evaporated *in vacuo*. The crude product was chromatographed on a silica-gel column using a mixed solvent of ethyl acetate and *n*-hexane (1:1) as eluent, and recrystallized from dichloromethane/*n*-hexane (1:20, *v*/*v*) to give as a crystalline solid in 70% yield (m.p. 424 K). IR (KBr pellet): 2926, 1600, 1504, 1235, 1126, 996 and 735 cm^-1. 1^H NMR (CDCl_3_): *δ* 7.54 (d, 4H, Ar-*H*), 7.06–6.91 (m, 8H, Ar-*H*), 5.06 (s, 4H, ArC*H*_2_O), 4.12 (t, 4H, ArOC*H*_2_CH_2_OCH_2_), 3.88 (t, 4H, ArOCH_2_C*H*_2_OCH_2_) and 3.70 (t, 4H, ArOCH_2_CH_2_OC*H*_2_).

### Refinement

(type here to add refinement details)

### Crystal data

**Table:**

C_26_H_28_O_6_	*F*(000) = 928
*M**_r_* = 436.48	*D*_x_ = 1.313 Mg m^−^^3^
Monoclinic, *P*2_1_/*c*	Mo *K*α radiation, λ = 0.71073 Å
Hall symbol: -P 2ybc	Cell parameters from 3896 reflections
*a* = 12.348 (3) Å	θ = 1.7–25.0°
*b* = 18.908 (4) Å	µ = 0.09 mm^−^^1^
*c* = 9.824 (2) Å	*T* = 293 K
β = 105.70 (3)°	Plate, colorless
*V* = 2208.0 (8) Å^3^	0.25 × 0.20 × 0.10 mm
*Z* = 4	

### Data collection

**Table:**

Bruker SMART CCD area-detector diffractometer	2654 reflections with *I* > 2σ(*I*)
Radiation source: fine-focus sealed tube	*R*_int_ = 0.057
graphite	θ_max_ = 25.1°, θ_min_ = 1.7°
ω scans	*h* = −14→14
4142 measured reflections	*k* = 0→22
3896 independent reflections	*l* = −11→0

### Refinement

**Table:**

Refinement on *F*^2^	Primary atom site location: structure-invariant direct methods
Least-squares matrix: full	Secondary atom site location: difference Fourier map
*R*[*F*^2^ > 2σ(*F*^2^)] = 0.041	Hydrogen site location: inferred from neighbouring sites
*wR*(*F*^2^) = 0.116	H-atom parameters constrained
*S* = 1.02	*w* = 1/[σ^2^(*F*_o_^2^) + (0.0672*P*)^2^ + 0.0279*P*] where *P* = (*F*_o_^2^ + 2*F*_c_^2^)/3
3896 reflections	(Δ/σ)_max_ < 0.001
289 parameters	Δρ_max_ = 0.14 e Å^−^^3^
0 restraints	Δρ_min_ = −0.18 e Å^−^^3^

### Special details

**Table:**

Geometry. All e.s.d.'s (except the e.s.d. in the dihedral angle between two l.s. planes) are estimated using the full covariance matrix. The cell e.s.d.'s are taken into account individually in the estimation of e.s.d.'s in distances, angles and torsion angles; correlations between e.s.d.'s in cell parameters are only used when they are defined by crystal symmetry. An approximate (isotropic) treatment of cell e.s.d.'s is used for estimating e.s.d.'s involving l.s. planes.
Refinement. Refinement of *F*^2^ against ALL reflections. The weighted *R*-factor *wR* and goodness of fit *S* are based on *F*^2^, conventional *R*-factors *R* are based on *F*, with *F* set to zero for negative *F*^2^. The threshold expression of *F*^2^ > σ(*F*^2^) is used only for calculating *R*-factors(gt) *etc*. and is not relevant to the choice of reflections for refinement. *R*-factors based on *F*^2^ are statistically about twice as large as those based on *F*, and *R*- factors based on ALL data will be even larger.

### Fractional atomic coordinates and isotropic or equivalent isotropic displacement parameters (Å^2^)

**Table:**

	*x*	*y*	*z*	*U*_iso_*/*U*_eq_	
O1	−0.14749 (11)	0.20218 (7)	1.10961 (13)	0.0498 (3)	
O2	−0.28741 (11)	0.11040 (7)	0.97137 (13)	0.0520 (4)	
O3	−0.17603 (11)	0.00949 (7)	0.83350 (15)	0.0568 (4)	
O4	−0.00678 (11)	0.00265 (9)	0.68633 (14)	0.0624 (4)	
O5	0.23169 (10)	0.03178 (7)	0.73303 (12)	0.0474 (3)	
O6	0.29941 (11)	0.14286 (7)	0.87761 (14)	0.0552 (4)	
C1	−0.23684 (15)	0.18893 (10)	1.16215 (19)	0.0446 (4)	
C2	−0.25554 (17)	0.21956 (11)	1.2812 (2)	0.0538 (5)	
H2	−0.2057	0.2534	1.3313	0.065*	
C3	−0.34797 (19)	0.20021 (12)	1.3264 (2)	0.0626 (6)	
H3	−0.3593	0.2204	1.4077	0.075*	
C4	−0.42279 (19)	0.15159 (12)	1.2525 (2)	0.0639 (6)	
H4	−0.4854	0.1393	1.2828	0.077*	
C5	−0.40553 (17)	0.12059 (11)	1.1322 (2)	0.0571 (5)	
H5	−0.4568	0.0876	1.0820	0.069*	
C6	−0.31315 (15)	0.13823 (10)	1.08660 (18)	0.0449 (4)	
C7	−0.33649 (16)	0.04425 (10)	0.9193 (2)	0.0509 (5)	
H7A	−0.4178	0.0485	0.8889	0.061*	
H7B	−0.3169	0.0088	0.9933	0.061*	
C8	−0.29280 (16)	0.02303 (12)	0.7974 (2)	0.0557 (5)	
H8A	−0.3324	−0.0192	0.7546	0.067*	
H8B	−0.3098	0.0603	0.7271	0.067*	
C9	−0.11025 (18)	0.06755 (10)	0.8132 (2)	0.0567 (5)	
H9A	−0.1033	0.1014	0.8893	0.068*	
H9B	−0.1464	0.0910	0.7247	0.068*	
C10	0.00358 (16)	0.04204 (11)	0.8106 (2)	0.0532 (5)	
H10A	0.0533	0.0820	0.8129	0.064*	
H10B	0.0354	0.0127	0.8927	0.064*	
C11	0.07916 (16)	−0.04707 (10)	0.6910 (2)	0.0551 (5)	
H11A	0.0465	−0.0882	0.6360	0.066*	
H11B	0.1099	−0.0623	0.7881	0.066*	
C12	0.17270 (15)	−0.02012 (10)	0.6366 (2)	0.0480 (5)	
H12A	0.2229	−0.0585	0.6292	0.058*	
H12B	0.1430	0.0006	0.5436	0.058*	
C13	0.31566 (15)	0.06729 (10)	0.69757 (18)	0.0426 (4)	
C14	0.36356 (17)	0.04880 (11)	0.5914 (2)	0.0541 (5)	
H14	0.3391	0.0084	0.5380	0.065*	
C15	0.44755 (18)	0.08973 (12)	0.5636 (2)	0.0617 (6)	
H15	0.4794	0.0768	0.4916	0.074*	
C16	0.48412 (16)	0.14911 (12)	0.6415 (2)	0.0588 (6)	
H16	0.5405	0.1767	0.6223	0.071*	
C17	0.43756 (16)	0.16805 (11)	0.7484 (2)	0.0530 (5)	
H17	0.4633	0.2083	0.8018	0.064*	
C18	0.35325 (14)	0.12816 (10)	0.77722 (19)	0.0427 (4)	
C19	0.30700 (16)	0.21254 (10)	0.9329 (2)	0.0521 (5)	
H19A	0.2975	0.2468	0.8571	0.063*	
H19B	0.3801	0.2199	0.9994	0.063*	
C20	0.21604 (15)	0.22107 (9)	1.00562 (19)	0.0457 (4)	
C21	0.16951 (16)	0.28661 (10)	1.0145 (2)	0.0501 (5)	
H21	0.1968	0.3260	0.9777	0.060*	
C22	0.08275 (17)	0.29458 (10)	1.0775 (2)	0.0503 (5)	
H22	0.0532	0.3394	1.0833	0.060*	
C23	0.03947 (16)	0.23753 (10)	1.1316 (2)	0.0492 (5)	
C24	0.08909 (18)	0.17237 (10)	1.1269 (2)	0.0565 (5)	
H24	0.0634	0.1333	1.1664	0.068*	
C25	0.17552 (17)	0.16431 (10)	1.0652 (2)	0.0548 (5)	
H25	0.2073	0.1198	1.0635	0.066*	
C26	−0.05795 (17)	0.24454 (11)	1.1932 (2)	0.0574 (5)	
H26A	−0.0373	0.2284	1.2905	0.069*	
H26B	−0.0813	0.2936	1.1914	0.069*	

### Atomic displacement parameters (Å^2^)

**Table:**

	*U*^11^	*U*^22^	*U*^33^	*U*^12^	*U*^13^	*U*^23^
O1	0.0493 (7)	0.0561 (8)	0.0464 (7)	−0.0046 (6)	0.0169 (6)	−0.0083 (6)
O2	0.0533 (8)	0.0572 (8)	0.0480 (7)	−0.0055 (6)	0.0178 (6)	−0.0082 (6)
O3	0.0508 (8)	0.0470 (8)	0.0751 (9)	0.0046 (6)	0.0214 (7)	0.0069 (7)
O4	0.0447 (7)	0.0944 (11)	0.0434 (7)	0.0134 (8)	0.0038 (6)	−0.0129 (7)
O5	0.0492 (7)	0.0495 (7)	0.0453 (7)	−0.0086 (6)	0.0156 (6)	−0.0089 (6)
O6	0.0656 (9)	0.0480 (8)	0.0600 (8)	−0.0131 (7)	0.0305 (7)	−0.0144 (6)
C1	0.0464 (10)	0.0450 (11)	0.0425 (10)	0.0107 (8)	0.0121 (9)	0.0034 (8)
C2	0.0593 (12)	0.0511 (12)	0.0526 (12)	0.0106 (10)	0.0179 (10)	−0.0054 (9)
C3	0.0652 (13)	0.0716 (14)	0.0568 (13)	0.0154 (12)	0.0268 (11)	−0.0045 (11)
C4	0.0554 (12)	0.0785 (16)	0.0661 (14)	0.0117 (12)	0.0305 (11)	0.0083 (12)
C5	0.0474 (11)	0.0663 (14)	0.0587 (13)	0.0043 (10)	0.0163 (10)	0.0020 (11)
C6	0.0449 (10)	0.0507 (11)	0.0406 (10)	0.0109 (9)	0.0139 (8)	0.0031 (9)
C7	0.0446 (10)	0.0529 (12)	0.0528 (11)	−0.0026 (9)	0.0092 (9)	−0.0038 (9)
C8	0.0479 (11)	0.0608 (12)	0.0550 (12)	−0.0029 (10)	0.0080 (9)	−0.0118 (10)
C9	0.0617 (13)	0.0452 (11)	0.0686 (13)	0.0005 (10)	0.0269 (11)	0.0013 (10)
C10	0.0517 (11)	0.0608 (13)	0.0468 (11)	−0.0001 (10)	0.0127 (9)	−0.0047 (10)
C11	0.0494 (11)	0.0522 (12)	0.0626 (12)	−0.0069 (10)	0.0129 (10)	−0.0170 (10)
C12	0.0466 (11)	0.0488 (11)	0.0455 (10)	0.0017 (9)	0.0070 (9)	−0.0105 (9)
C13	0.0396 (10)	0.0469 (11)	0.0412 (10)	0.0054 (8)	0.0107 (8)	0.0063 (8)
C14	0.0577 (12)	0.0583 (12)	0.0500 (11)	0.0051 (10)	0.0209 (10)	−0.0012 (10)
C15	0.0609 (13)	0.0730 (15)	0.0609 (13)	0.0107 (12)	0.0328 (11)	0.0073 (12)
C16	0.0469 (11)	0.0640 (14)	0.0714 (14)	0.0053 (10)	0.0261 (11)	0.0164 (12)
C17	0.0442 (10)	0.0521 (11)	0.0617 (13)	−0.0023 (9)	0.0130 (10)	0.0027 (10)
C18	0.0406 (10)	0.0445 (10)	0.0440 (10)	0.0028 (8)	0.0133 (8)	0.0031 (8)
C19	0.0529 (12)	0.0452 (11)	0.0591 (12)	−0.0097 (9)	0.0168 (10)	−0.0106 (9)
C20	0.0462 (10)	0.0429 (10)	0.0452 (10)	−0.0079 (8)	0.0073 (9)	−0.0096 (8)
C21	0.0584 (12)	0.0397 (10)	0.0499 (11)	−0.0091 (9)	0.0106 (9)	−0.0050 (9)
C22	0.0595 (12)	0.0394 (10)	0.0496 (11)	−0.0002 (9)	0.0106 (10)	−0.0082 (9)
C23	0.0516 (11)	0.0464 (11)	0.0488 (11)	−0.0063 (9)	0.0121 (9)	−0.0148 (9)
C24	0.0676 (13)	0.0405 (11)	0.0681 (14)	−0.0068 (10)	0.0296 (11)	−0.0043 (10)
C25	0.0629 (13)	0.0379 (11)	0.0679 (13)	0.0010 (9)	0.0250 (11)	−0.0031 (9)
C26	0.0605 (13)	0.0522 (12)	0.0618 (13)	−0.0069 (10)	0.0205 (11)	−0.0191 (10)

### Geometric parameters (Å, °)

**Table:**

O1—C1	1.362 (2)	C11—C12	1.488 (3)
O1—C26	1.431 (2)	C11—H11A	0.9700
O2—C6	1.362 (2)	C11—H11B	0.9700
O2—C7	1.423 (2)	C12—H12A	0.9700
O3—C9	1.412 (2)	C12—H12B	0.9700
O3—C8	1.412 (2)	C13—C14	1.376 (3)
O4—C10	1.406 (2)	C13—C18	1.398 (3)
O4—C11	1.409 (2)	C14—C15	1.379 (3)
O5—C13	1.357 (2)	C14—H14	0.9300
O5—C12	1.420 (2)	C15—C16	1.365 (3)
O6—C18	1.359 (2)	C15—H15	0.9300
O6—C19	1.418 (2)	C16—C17	1.374 (3)
C1—C2	1.380 (3)	C16—H16	0.9300
C1—C6	1.407 (3)	C17—C18	1.376 (3)
C2—C3	1.381 (3)	C17—H17	0.9300
C2—H2	0.9300	C19—C20	1.494 (3)
C3—C4	1.365 (3)	C19—H19A	0.9700
C3—H3	0.9300	C19—H19B	0.9700
C4—C5	1.386 (3)	C20—C21	1.379 (3)
C4—H4	0.9300	C20—C25	1.380 (3)
C5—C6	1.375 (3)	C21—C22	1.383 (3)
C5—H5	0.9300	C21—H21	0.9300
C7—C8	1.495 (3)	C22—C23	1.373 (3)
C7—H7A	0.9700	C22—H22	0.9300
C7—H7B	0.9700	C23—C24	1.382 (3)
C8—H8A	0.9700	C23—C26	1.491 (3)
C8—H8B	0.9700	C24—C25	1.371 (3)
C9—C10	1.493 (3)	C24—H24	0.9300
C9—H9A	0.9700	C25—H25	0.9300
C9—H9B	0.9700	C26—H26A	0.9700
C10—H10A	0.9700	C26—H26B	0.9700
C10—H10B	0.9700		
			
C1—O1—C26	117.71 (14)	O5—C12—C11	107.74 (14)
C6—O2—C7	117.76 (14)	O5—C12—H12A	110.2
C9—O3—C8	114.27 (16)	C11—C12—H12A	110.2
C10—O4—C11	115.74 (15)	O5—C12—H12B	110.2
C13—O5—C12	117.39 (13)	C11—C12—H12B	110.2
C18—O6—C19	118.25 (14)	H12A—C12—H12B	108.5
O1—C1—C2	125.65 (18)	O5—C13—C14	125.71 (17)
O1—C1—C6	114.90 (15)	O5—C13—C18	115.11 (15)
C2—C1—C6	119.44 (18)	C14—C13—C18	119.17 (17)
C1—C2—C3	120.2 (2)	C13—C14—C15	120.5 (2)
C1—C2—H2	119.9	C13—C14—H14	119.7
C3—C2—H2	119.9	C15—C14—H14	119.7
C4—C3—C2	120.5 (2)	C16—C15—C14	120.18 (19)
C4—C3—H3	119.8	C16—C15—H15	119.9
C2—C3—H3	119.8	C14—C15—H15	119.9
C3—C4—C5	120.0 (2)	C15—C16—C17	119.98 (19)
C3—C4—H4	120.0	C15—C16—H16	120.0
C5—C4—H4	120.0	C17—C16—H16	120.0
C6—C5—C4	120.5 (2)	C16—C17—C18	120.7 (2)
C6—C5—H5	119.7	C16—C17—H17	119.6
C4—C5—H5	119.7	C18—C17—H17	119.6
O2—C6—C5	125.33 (18)	O6—C18—C17	125.69 (17)
O2—C6—C1	115.35 (16)	O6—C18—C13	114.89 (15)
C5—C6—C1	119.32 (17)	C17—C18—C13	119.41 (17)
O2—C7—C8	108.22 (16)	O6—C19—C20	107.65 (15)
O2—C7—H7A	110.1	O6—C19—H19A	110.2
C8—C7—H7A	110.1	C20—C19—H19A	110.2
O2—C7—H7B	110.1	O6—C19—H19B	110.2
C8—C7—H7B	110.1	C20—C19—H19B	110.2
H7A—C7—H7B	108.4	H19A—C19—H19B	108.5
O3—C8—C7	114.40 (16)	C21—C20—C25	117.85 (18)
O3—C8—H8A	108.7	C21—C20—C19	120.50 (17)
C7—C8—H8A	108.7	C25—C20—C19	121.65 (17)
O3—C8—H8B	108.7	C20—C21—C22	120.86 (18)
C7—C8—H8B	108.7	C20—C21—H21	119.6
H8A—C8—H8B	107.6	C22—C21—H21	119.6
O3—C9—C10	109.36 (16)	C23—C22—C21	121.17 (18)
O3—C9—H9A	109.8	C23—C22—H22	119.4
C10—C9—H9A	109.8	C21—C22—H22	119.4
O3—C9—H9B	109.8	C22—C23—C24	117.69 (18)
C10—C9—H9B	109.8	C22—C23—C26	121.89 (18)
H9A—C9—H9B	108.2	C24—C23—C26	120.41 (18)
O4—C10—C9	108.74 (17)	C25—C24—C23	121.26 (19)
O4—C10—H10A	109.9	C25—C24—H24	119.4
C9—C10—H10A	109.9	C23—C24—H24	119.4
O4—C10—H10B	109.9	C24—C25—C20	121.07 (19)
C9—C10—H10B	109.9	C24—C25—H25	119.5
H10A—C10—H10B	108.3	C20—C25—H25	119.5
O4—C11—C12	114.25 (17)	O1—C26—C23	107.49 (15)
O4—C11—H11A	108.7	O1—C26—H26A	110.2
C12—C11—H11A	108.7	C23—C26—H26A	110.2
O4—C11—H11B	108.7	O1—C26—H26B	110.2
C12—C11—H11B	108.7	C23—C26—H26B	110.2
H11A—C11—H11B	107.6	H26A—C26—H26B	108.5
			
C26—O1—C1—C2	8.2 (3)	C13—C14—C15—C16	0.1 (3)
C26—O1—C1—C6	−170.39 (16)	C14—C15—C16—C17	0.3 (3)
O1—C1—C2—C3	−178.12 (17)	C15—C16—C17—C18	−0.7 (3)
C6—C1—C2—C3	0.5 (3)	C19—O6—C18—C17	17.7 (3)
C1—C2—C3—C4	−1.2 (3)	C19—O6—C18—C13	−161.32 (17)
C2—C3—C4—C5	0.8 (3)	C16—C17—C18—O6	−178.25 (18)
C3—C4—C5—C6	0.2 (3)	C16—C17—C18—C13	0.7 (3)
C7—O2—C6—C5	−20.6 (3)	O5—C13—C18—O6	−0.4 (2)
C7—O2—C6—C1	159.34 (16)	C14—C13—C18—O6	178.72 (16)
C4—C5—C6—O2	179.09 (18)	O5—C13—C18—C17	−179.49 (16)
C4—C5—C6—C1	−0.9 (3)	C14—C13—C18—C17	−0.3 (3)
O1—C1—C6—O2	−0.7 (2)	C18—O6—C19—C20	163.38 (15)
C2—C1—C6—O2	−179.42 (16)	O6—C19—C20—C21	−151.26 (17)
O1—C1—C6—C5	179.28 (16)	O6—C19—C20—C25	28.5 (2)
C2—C1—C6—C5	0.5 (3)	C25—C20—C21—C22	−1.9 (3)
C6—O2—C7—C8	−177.71 (15)	C19—C20—C21—C22	177.84 (17)
C9—O3—C8—C7	−95.6 (2)	C20—C21—C22—C23	−0.7 (3)
O2—C7—C8—O3	64.9 (2)	C21—C22—C23—C24	3.0 (3)
C8—O3—C9—C10	−162.52 (16)	C21—C22—C23—C26	−176.78 (18)
C11—O4—C10—C9	−156.07 (16)	C22—C23—C24—C25	−2.6 (3)
O3—C9—C10—O4	69.2 (2)	C26—C23—C24—C25	177.11 (19)
C10—O4—C11—C12	−93.9 (2)	C23—C24—C25—C20	0.0 (3)
C13—O5—C12—C11	−175.06 (15)	C21—C20—C25—C24	2.2 (3)
O4—C11—C12—O5	68.5 (2)	C19—C20—C25—C24	−177.50 (19)
C12—O5—C13—C14	−13.4 (3)	C1—O1—C26—C23	166.99 (15)
C12—O5—C13—C18	165.73 (15)	C22—C23—C26—O1	118.0 (2)
O5—C13—C14—C15	179.01 (17)	C24—C23—C26—O1	−61.7 (2)
C18—C13—C14—C15	−0.1 (3)		

### Hydrogen-bond geometry (Å, °)

**Table:**

*D*—H···*A*	*D*—H	H···*A*	*D*···*A*	*D*—H···*A*
C9—H9A···O2	0.97	2.62	3.114 (2)	112
C10—H10A···O5	0.97	2.70	3.117 (2)	106
C2—H2···O1^i^	0.93	2.76	3.471 (2)	134
C7—H7B···O5^ii^	0.97	2.72	3.607 (2)	153
C11—H11B···O1^ii^	0.97	2.82	3.501 (3)	128
C11—H11B···O2^ii^	0.97	2.90	3.798 (3)	154
C12—H12B···O4^iii^	0.97	2.42	3.294 (3)	149
C25—H25···O3^ii^	0.93	2.71	3.433 (2)	135
C12—H12A···Cg1^ii^	0.97	2.76	3.47	138
C21—H21···Cg2^i^	0.93	2.97	3.47	115
C26—H26A···Cg3^i^	0.97	3.06	3.83	138

Symmetry codes: (i) *x*, −*y*+1/2, *z*+1/2; (ii) −*x*, −*y*, −*z*+2; (iii) −*x*, −*y*, −*z*+1.
